# Assessing the Impact of Lean Healthcare on Inpatient Care: A Systematic Review

**DOI:** 10.3390/ijerph17155609

**Published:** 2020-08-04

**Authors:** Carlos Zepeda-Lugo, Diego Tlapa, Yolanda Baez-Lopez, Jorge Limon-Romero, Sinue Ontiveros, Armando Perez-Sanchez, Guilherme Tortorella

**Affiliations:** 1 Facultad de Ingeniería, Arquitectura y Diseño, Universidad Autónoma de Baja California, Ensenada 22860, Mexico; czepeda@uabc.edu.mx (C.Z.-L.); jorge.limon@uabc.edu.mx (J.L.-R.); 2 Facultad de Ciencias de la Ingeniería, Administrativas y Sociales, Universidad Autónoma de Baja California, Tecate 21460, Mexico; sinue.ontiveros@uabc.edu.mx; 3 Facultad de Ciencias de la Ingeniería y Tecnología, Universidad Autónoma de Baja California, Tijuana 22260, Mexico; armando.perez.sanchez@uabc.edu.mx; 4 Department of Systems and Production Engineering, Universidade Federal de Santa Catarina, Florianópolis 88040, Brazil; gtortorella@bol.com.br

**Keywords:** inpatient care, lean healthcare, efficiency, patient flow, systematic review, length of stay, turnaround time, turnover time, on-time starts, boarding time, readmission rate, discharge time

## Abstract

Healthcare services are facing challenges in increasing their efficiency, quality of care, and coping with surges in demand. To this end, some hospitals have implemented lean healthcare. The aim of this systematic review is to evaluate the effects of lean healthcare (LH) interventions on inpatient care and determine whether patient flow and efficiency outcomes improve. The review was performed according to PRISMA. We used six databases to search for studies published from 2002 to 2019. Out of 5732 studies, 39 measuring one or more defined outcomes were included. Hospital length of stay (LOS) was measured in 23 studies, 16 of which reported a reduction, turnover time (TOT) decreased in six out of eight studies, while the turnaround time (TAT) and on-time starts (OTS) improved in all five and seven studies, respectively. Moreover, eight out of nine studies reported an earlier discharge time, and the boarding time decreased in all four cases. Meanwhile, the readmission rate did not increase in all nine studies. Lastly, staff and patient satisfaction improved in all eight studies. Our findings show that by focusing on reducing non-value-added activities, LH contributed to improving patient flow and efficiency within inpatient care.

## 1. Introduction

In addition to the constant demand to improve their quality of care, hospitals are facing challenges in increasing their efficiency [1], reducing costs [2], and coping with surges in demand, while providing greater value to patients. Inefficiencies such as inadequate resource utilization and poor patient flow, might contribute to care delays and overcrowding, therefore affecting the safety of patients, staff/patient satisfaction, and the overall care quality [3,4]. Patient flow is the movement of patients through care settings [5]. It encompasses the physical resources, medical care, and internal systems required to get patients from the admission to the discharge while preserving quality and patient/staff satisfaction [6]. Both inpatient and outpatient care present opportunities to increase efficiency [7]. Hence, efficiency measures and performance indicators are paramount in the survival of healthcare systems [8]. Some measures used in outpatient care include emergency department length of stay (LOS) [9,10], the waiting time to see a healthcare professional [11,12], the waiting time for treatment [13], the waiting time for triage [14], and patients left without being seen [15,16], among others. Conversely, some common indicators within inpatient care are the hospital length of stay (LOS) [17,18], boarding time [3,19], and discharge order time [19,20]. Likewise, major areas of inefficiency that hospitals are trying to reduce are present in the perioperative workflow [21]. Hence, additional indicators include turnover time (TOT) [8,21,22], turnaround time (TAT) [23,24,25], and on-time starts (OTS) [8,26,27]. A related outcome is the readmission or revisit rate [28,29]. Within inpatient care, hospital overcrowding has become a widespread problem, with constrained bed capacity and admission bottlenecks having negative impacts on quality and safety. Inpatient hospital services represent 20% of medicare spending, whereas outpatients account for 8% [30].

The patient admission scheduling problem has been revisited from different approaches. Through operations research, the authors in [31] propose a model considering LOS, admission, discharge time, and constraints on the utilization of operating rooms for patients requiring a surgery. Similarly, an algorithm is proposed to scheduling patient admissions more efficiently, taking into account the medical needs of the patients as well as their preferences [32]. In [33], the authors try to maximize patient satisfaction, taking into account the expected LOS and the room overcrowding risk. 

In an attempt to deal with both cost issues and quality, healthcare providers have been looking outside the healthcare area for guidance and inspiration [34]. To increase their efficiency, hospitals are implementing lean healthcare (LH) in their processes, with a focus on eliminating waste [35] while increasing the value for patients. Healthcare value has different definitions [36]. In this study, such values are considered “activities that enhance the quality of healthcare and promote patient well-being so as to achieve better outcomes” [37]. In connection with this, LH divide activities into either non-value added (NVA) or value added (VA) [38]; the VA activities contribute to fulfilling patient needs, whereas the NVA activities use unnecessary space, time or resources and do not meet patient needs [38,39]. LH contributes to exposing NVA activities and taking action to reduce or eliminate them [40]. Similarly, waste is anything other than the minimum quantity of space, equipment, or staff time that is necessary to add value to a service or product [41]. The term “lean” initiates from the Toyota Production System (TPS) [42], which aimed at increasing processes efficiency. The TPS entered the medical sector in the early 2000 s commonly known as lean healthcare [43,44]. Applying the TPS was recognized as an effective strategy to improve outcomes and lower costs by incrementing the efficiency of hospital-based clinical care [45]. In the U.S., a survey found that about 70% of hospitals implement LH or similar approaches [46]. Since its introduction, LH has been implemented in virtually all hospital departments, including cardiology, surgery, and intensive care units (ICUs) [21,28,29,47,48,49,50,51,52,53].

LH is not free from difficulties, including adjustments in transferring the principles and tools to a new setting [25], as well as methodological restrictions at the implementation phase [54,55]. Different authors have summarized the effects of LH through systematic reviews (SR) with different approaches, such as LH within emergency departments [56], quality improvements in surgery [57], lean-six sigma in surgery [58], lean-six sigma in radiology [59], and lean-six sigma in the healthcare industry [60], whereas others have focused on care efficiency measures [61], contextual aspects and change mechanisms [62], lean facilitators [63], and the positive impacts of LH [64]. Additionally, reviews on LH provide thematic analyses [65], updates [44], and operational definitions [66]. Still others are focused on hospital waste management [67], the choosing wisely approach [68], sustainability [69], leadership and management [70], and safety and patient care [71]. To the best of our knowledge there is not a SR focusing on inpatient care and outcomes related to patient flow and efficiency. Complementarily, different authors have proposed models analyzing the relationship between LH and performance outcomes using structural equation modelling [72,73,74,75] and confirmatory factor analysis [76].

Notwithstanding these studies, research on the effect of LH on efficiency and patient flow within inpatient care still remains in its early stages. To address this gap, our research aims to classify, organize, and summarize evidence regarding the effects of LH on efficiency and patient flow outcomes within inpatient care. To contribute to the body of knowledge on LH, we conducted a systematic review to determine whether LOS, TOT, TAT, OTS, boarding time, discharge times, and readmission rates are improved with a LH intervention. In addition, we reviewed the changes in satisfaction of patient and staff as secondary outcomes. The remainder of this paper is organized as follows: Section 2 describes the methodology adopted for this systematic review. The summary of results is presented in Section 3. Section 4 widely discusses our results. Finally, limitations and conclusions are presented in Section 5 and Section 6, respectively. 

## 2. Methods

**Figure 1:**
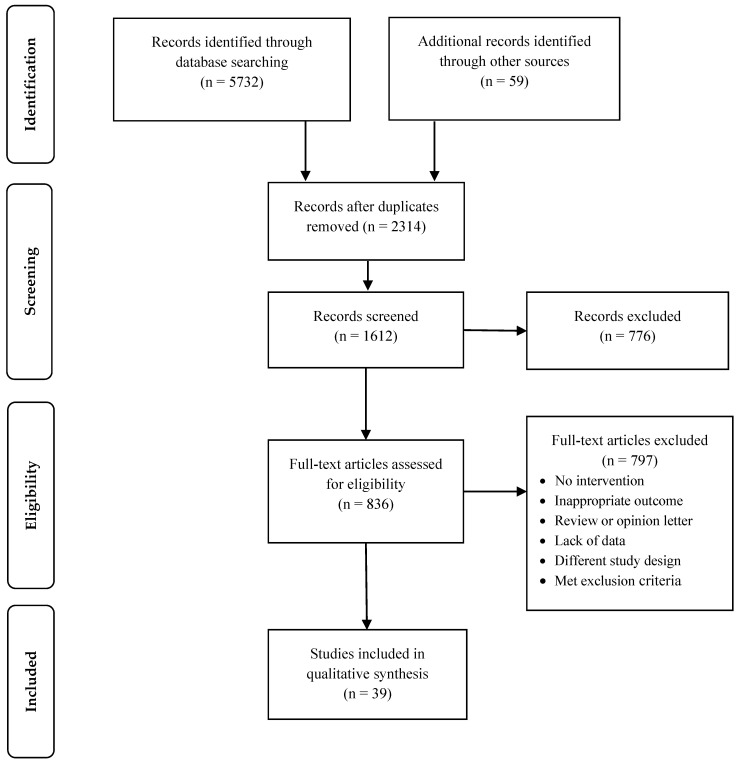
Preferred Reporting Items for Systematic Reviews and Meta-Analyses (PRISMA) flow chart.

For this systematic review we registered a protocol on the International Prospective Register of Systematic Reviews (PROSPERO; Ref CRD42019134287). The review was conducted according to the Preferred Reporting Items for Systematic Reviews and Meta-Analyses (PRISMA) [77,78]. Figure 1 presents the flowchart of the stages involved in the selection process, while the resulting PRISMA checklist summarizes all the requirements covered (see online Supplementary Table S1). The subsequent subsections discuss the methodology.

### 2.1. Data Source and Search Strategy

For the search we used the following databases: PubMed-Medline, CINAHL, The Cochrane Library, Scopus, Web of Science, and Ebsco. In addition, we searched for grey literature on OpenGrey, Grey Literature Report, Google Scholar, and ProQuest. An initial search was performed to develop a search strategy based on the Peer Review of Electronic Search Strategies (PRESS) [79]. The ultimate search strategy is depicted in the Supplementary Data S1. We utilized components of the Effective Practice and Organisation of Care (EPOC) group’s search strategy, combined with selected MeSH terms and free text terms of PICOS elements (population, intervention, comparator, outcome, and study design). We collected studies published between 2002 and 2019 in English. Likewise, we examined the references of the retrieved articles to look for additional studies. We re-ran the search before the final analysis.

### 2.2. Study Selection

We selected studies whose main intervention was LH (also named as TPS or lean) within inpatient care from both the private and public sectors, studies improving patient flow, and studies providing sufficient data (in the study or by email). In addition, studies addressing similar interventions such as six sigma, rapid improvement event (RIE), or Baystate Patient Progress Initiative (BPPI) were also selected. We classified interventions as implementation strategies according to the taxonomy of the Cochrane EPOC Group [80], particularly in the continuous quality improvement subcategory. Conversely, the exclusion criteria for studies were defined as follows: studies considering ambulatory times, studies not involving a patient flow-related outcome (e.g., medical device efficiency, supplier efficiency, and medical device manufacturer efficiency), studies lacking data, and literature on pharmacologic interventions. We searched for randomized controlled trials (RCTs), controlled before–after, and quasi-RCT studies. Additionally, we included a case-control, cohort, and pre–post studies. Cross-sectional studies, abstracts, surveys, and opinion papers were excluded.

We categorized the main outcomes as utilization of services, access to services, and healthcare resources use [81]. For the former, we reviewed the changes in the length of stay (LOS) for patients admitted (i.e., the time from arrival to bed to discharge from the hospital). For the perioperative process, we reviewed changes in the outcome on-time starts (OTS), measured by the change of the percentage of starting a procedure on time; the turnover time (TOT), measured as the interval in minutes between patient departure and the arrival of the subsequent patient to the OR; and turnaround time (TAT), measured as the interval in minutes between the conclusion of surgical dressing and surgical incision of the subsequent.

As for access to services, we analyzed the boarding time, measured as the time patients spent since the decision to be admitted until being assigned to a bed, and the discharge order time, measured as the change in the percentage of early patient discharges or discharge orders before noon. Finally, we also reviewed the changes in readmission or re-visit rates to the hospital. As secondary outcomes, we reviewed the changes in satisfaction of patient and staff.

### 2.3. Data Extraction, Analysis and Synthesis

Each study was independently screened by two reviewers to identify the title, abstract, and keywords. The rate of disagreements was 14% and was solved by means of discussion. Then, two reviewers retrieved and assessed the full texts of the relevant studies based on the inclusion and exclusion criteria. If reviewers did not reach an agreement, then a third reviewer evaluated the study. The raw data were extracted by one reviewer and examined by a second reviewer; these data included the authors’ names, titles, publication years, settings, studies length, study designs, countries, participant demographics, intervention and control conditions, outcomes, and details for the risk of bias assessment. Finally, we used standardized forms to tabulate and organize the collected data. Given the heterogeneity of the studies in terms of their study designs (mainly observational and descriptive), settings, and outcomes, we were unable to pool the results and conduct a meta-analysis. Therefore, we provide a comparative summary of findings for the main outcomes using the measures of effect (i.e., the means, medians, or percentages) in the same way as they were reported.

### 2.4. Risk of Bias

The risk of bias was assessed by means of the Cochrane’s tool ROBINS-I (Risk Of Bias In Non-randomized Studies of Interventions) [82,83]. We used this tool because most of the research was observational and assessed the systematic difference between the results found from a non-randomized study of interventions (NRSI) and a pragmatic randomized trial [84]. The judgment criteria were comprised of five levels (no information, critical, serious, moderate, and low) for each of the seven bias domains covered by the ROBINS-I tool [82]. Two reviewers independently used the algorithm from ROBINS-I to reach an overall risk of bias (RoB) judgment for each study; if a difference persisted between the reviewers, then a third reviewer evaluated the research and came to an agreement.

## 3. Results

We found 5732 studies in the initial phase. Duplicates removal resulted in 2314 potentially pertinent papers. Then, in the screening phase, 776 studies were removed after applying the exclusion criteria. Moreover, 836 LH interventions underwent a full-text review. Yet, 797 studies were excluded for the reasons stated in Figure 1. Ultimately, the systematic review comprised 39 LH interventions. For settings, 21 of the LH interventions were conducted in the operating room/surgical units, whereas five focused on the emergency department (ED) and two on the intensive care units, among others. In addition, most of the studies were conducted in the U.S. (n = 27), Spain (n = 2), and the Netherlands (n = 2); similarly, early LH interventions seem to have arisen in 2004, yet there is an increase after 2011 (see online Supplementary Tables S2 and S3).

Sixteen studies informed a LOS decrease following LH interventions, with 105.85 days representing the longest reduction [85]. Conversely, only seven studies stated no variation after the interventions [20,29,86,87,88,89,90]. For turnover time (TOT), six studies confirmed a decrease, whereas only two studies reported no change [26,27]. Moreover, the turnaround time (TAT) decreased in the five studies that addressed it. Boarding time was only evaluated in four LH interventions, with better results in all of them [3,19,29,91].

The readmission rate was evaluated in nine studies, with none reporting an increase. Seven of them showed no change, and two studies reported a reduction [90,92]. The outcome on-time starts (OTS), was measured in seven studies, all of them with positive results. For discharge order time, eight studies reported an earlier discharge time, while one study reported no change [93]. Table 1 shows the direction of the findings for the main outcomes. In addition, main outcomes, statistics, and descriptions (when available) are summarized in Table 2. Likewise, an extensive summary of findings is provided in Table S4 (online Supplementary Material), including five studies that fulfilled our requests for information. Interestingly, only eight studies reported a measure of patient satisfaction and staff satisfaction respectively, all of them reported an improvement after the intervention. Other important outcomes reported included the percentage of day of surgery cancellations [8], reduction in surgery times [94], reduction of time to surgery [95], reduction of unnecessary instruments delivered to the OR [94,96], and savings in nursing time [97]. Additional measures of efficiency after LH intervention included an increase in capacity for extra patients [97], additional ED bed hours per day [91], an increase in capacity of open beds per day [19,98], a reduction in transfer due to lack of beds [29], improved OR utilization [8,26,27], and an increase in surgical admissions receiving appropriate perioperative antibiotics [86]. Finally, mortality was measured in six studies, four of which reported no change after LH intervention [17,18,28,29], while two studies reported a decreased [87,98].

We found 25 interventions of lean and six sigma (LSS) within inpatient care, predominantly showing positive results. For work teams, 33 out of 39 interventions used multidisciplinary teams, most of these studies provided positive results in their outcomes. Meeting organizational, regional or national standards/targets for the reported outcome was only discussed in 9 out of 39 studies. Regarding the types of studies, 34 were pre–post studies, among which two used controls. Meanwhile, the remaining five were cohorts. None of the research involved RCTs. Finally, in terms of risk of bias, 28 interventions were assessed as moderate and 11 as serious (see online Supplementary Materials, Table S5).

## 4. Discussion

The outbreak of COVID-19 added more pressure to healthcare organizations, who face an ongoing challenge to improve efficiency and meet an increasing demand for high quality of care and lower costs. The objective of this systematic review was to evaluate the effects of lean healthcare interventions on efficiency and patient flow outcomes within inpatient care. Six out of seven outcomes presented an overall improvement, while readmission rates did not increase after the LH intervention. An extensive summary of findings is provided in Table S4 (online Supplementary Materials).

### 4.1. Length of Stay

First, in 23 out of 39 studies, LOS was the most common process-related outcome. This finding is consistent with previous studies [44,111]. LOS is a general measure of hospital efficiency [86] and is commonly related to costs reductions when the LOS is reduced [99]. We found mixed results for LOS across the departments of the hospitals in which LH was implemented. For cardiology, two studies [47,99] reported a reduction, while one study [90] reported a non-significative reduction. Accordingly, for orthopedic and trauma, five studies showed a reduction in LOS [48,92,95,98,102]. Meanwhile, two studies did not reduced the LOS [87,88]. For both studies conducted in the ICU, one reduced the LOS [17], while the other reported no change in the ICU [29]. Interesting, when the main goal is to reduce infections within surgical units, LH contributed to reducing the LOS in two out of three studies [18,100], while one study [86] reported no change. Moreover, when LOS was reduced, other associated benefits were reported, such as cost reductions [17,95,99,100], a better return of investment [48], increased savings [47,90,93,98,102], or an earlier discharge order [3,19].

### 4.2. On-Time Starts, Turnover Time, and Turnaround Time

Major areas of inefficiency that hospitals try to reduce are found within the perioperative workflow [21]. The perioperative process includes the preoperative, operating room, and the postoperative departments, all of which have to run like a well-oiled machine to improve performance and achieve positive outcomes [112]. In this regard, preoperative throughput is an important element in achieving the perioperative goals for the first case’s on-time start (OTS) [105,113,114]. In this research, all seven studies improved their on-start times after LH intervention. This is consistent with the results of a previous study [109]. Thus, the preoperative use of LH leads to substantial improvements in OTS. An inefficient preoperative department can delay the start of surgery and impact the patient flow throughout the day [112], which can affect other outcomes, such as TOT and TAT, which are important performance parameters for the perioperative process. We found that LH led to reductions of TOT in six studies, the largest being a 50% reduction [104]. In contrast, two papers reported no significant changes in TOT, but other benefits, such as reductions from patient in room to procedure starting or OTS [26,27]. All four studies measuring the turnaround time (TAT) in the OR reported an improvement [21,23,24,101], with 20 min being the largest reduction [21]. Similar outcomes were obtained in the reduction of TAT for pathologists [25], as well as the medication TAT [18]. A comparable tool, the plan-do-study-act cycle [115], was also used to reduce the turnaround time [116]. Remarkably, all studies reporting an improvement in either OTS, TOT, or TAT, used multidisciplinary teams during the intervention. Other factors that might affect the perioperative process, include patient-related variables, personnel unavailability [117], surgeon variables [24,118], the workflow in the anesthesia preparation tasks [22,101,103], the type of the previous operative case (emergency), the waiting time for trays or patients arriving late [119], if a scheduled gap existed between the cases [21], patient family and social support [109], and even the weather [109].

### 4.3. Boarding Time, Early Discharge, and Readmission

Commonly targeted processes are the discharge and admission processes, due to the fact that these processes are almost always unnecessarily long and can have a large impact on the throughput of patients [111]. For the admission process, we found that the boarding time decreased in all four studies [3,19,29,91], in which 2.1 h was the longest reduction time, resulting in a boarding time of 5.5 h [19]. In the literature, boarding times ranged from 2 h (or less) to 24 h (or more), with medical/surgical patients experiencing shorter boarding times and behavioral patients experiencing longer boarding times [120]. It is recommended that boarding time frames not exceed 4 h in the interest of patient safety and quality of care [120].

The availability of beds is key to reducing the boarding time by improving inpatient discharge timing [121]. Therefore, focusing on the time of discharge may be the least disruptive and most effective way to address constrained bed capacity [20]. Both premature and delayed discharges not only worsen health outcomes but also increase costs. Premature discharges can lead to costly readmissions, while delayed discharges use up limited hospital resources [122]. Despite increasing the number of hospital beds will not solve completely the problems of overcrowding [122], it can affect patient health since delays in bed access compromise patient safety [123]. In addition, healthcare providers should plan their capacity to minimize the risks associated with occupancy rates exceeding 90% [124], i.e., bed shortages and higher rates of infection [122]. The factors affecting the discharge time include the hour of admission [107], preparation for discharge order [106], and the preparedness and cooperation of patients and their families [28].

Interestingly, none of the nine studies measuring readmission rates reported an increase after the intervention. Seven studies reported no change at 30 or 90 days and two studies reported a statistically significant reduction in readmission rates [90,92]. Using lean-six sigma, the readmission rate for heart failure patients was reduced up to 19.0% [90], while the US average heart failure readmission rate was 24.6% [125], and that in the UK was 17.8% by 2016 [126]. Recently, the emergency readmission within 30 days of discharge for all patients was found to be 14.4% in England [127]. To this end, healthcare organizations and governments have implemented financial incentives to improve the readmission rates, either to exceed the reduction of targets [128] or to avoid exceeding a threshold of emergency readmissions [129].

### 4.4. Patient and Staff Satisfaction

Patient satisfaction and experience are reported in only in 8 of 39 selected studies. This is contrary to our expectations since LH is considered a factor for improving the flow of patients and thus associated to increasing patient satisfaction [37,130,131,132,133]. Two studies used the Press Ganey assessment survey [89,91], while the other studies used self-developed surveys, including electronic surveys in combination with interviews [23,92,101].

Moreover, the literature suggests that healthcare professionals also notice LH benefits, such as an increase in their satisfaction [112,134,135,136,137] and empowerment [41,138]. Although satisfied patients and healthcare workers are prerequisites of sustainable high-quality care [139] and conversely disengaged healthcare workers are by far the main reason for lean failure [140], only 8 out of 39 studies measured staff satisfaction. With a hospital staff turnover rate of 17.8% by 2019 in the US [141] (which was reported to range from 15% to 36% in previous years) [142], this lack of evidence in the valuation of staff satisfaction following LH interventions suggests that generating the ideal staff experience has been absent from many LH transformations [140]. Among studies reporting staff satisfaction, the used instrument included the safety attitudes questionnaire (SAQ) and the operating room educational environment measure survey [21].

### 4.5. Perioperative Process

Improving patient flow in the perioperative environment is challenging but has positive implications for both staff members and the facility [101]. Thus, multidisciplinary perioperative teams [8], perioperative benchmark practices [48], and benchmarking meetings [95] have shown good results. Despite LSS research is abundant in the OR, there are minimal studies in a preoperative context [112]. LH and SS support the development of a clinical pathway [95,102] and thus reduce LOS [143]. The lack of relevant standardization was a common theme among pathways for patients or standard work for caregivers [48]. LOS, complications, patient satisfaction, education, and readmission have all been improved by the standardized pathways developed via LSS applications [26,143]. The purpose of such standards is to set expectations [144], care protocols, and staff roles [145], thereby reducing reliance on memory [144]. For this purpose, the standardization of care alongside evidence-based guidelines might enhance value in healthcare [146].

Operating rooms are linked to significant costs in most hospitals [147] but are potentially the most lucrative part of many healthcare systems [103]. With surgical care representing about a third of all healthcare spending [148], and with healthcare costs continuing to rise [149,150], healthcare enterprises are focusing on eliminating inpatient operational inefficiencies and waste to reduce unnecessary costs [93]. Thus, the OR represents an area with an opportunity to optimize work flow and supply use [151], and to maintaining an economically viable institution [22]. By applying process improvement methodologies, such as lean and six sigma, across an entire surgical suite, hospitals are attempting to improve efficiency [22]. These efforts appear to be a war on waste, which would be justified by the need to decrease costs that are not indispensable for patient care [152]. This intrinsic connection between LH and cost reductions/revenue increases was clear in about 43% of the studies (17 out of 39 studies). However, this number is still low, which suggests difficulties in interpreting such results into costs/savings, either due to a lack of personnel or training in this topic.

### 4.6. Lean Healthcare

We found 25 out of 39 studies combining lean with tools and principles of the six sigma methodology, suggesting that such integration offers a more robust approach to improving speed, quality and costs, increasing customer satisfaction, and maximizing shareholder value [153,154]. While lean focuses on reducing waste and NVA activities, six sigma focuses on reducing process variation by following the DMAIC approach (Define, Measure, Analyze, Improve and Control) and by using statistical tools. In this way, both methodologies complement each other. Lean-six sigma also provides useful frameworks to help hospital staff identify causes of delays in their own institutions [155]. This combination outperforms the use of only one methodology. Nevertheless, this integration tends to be composed of larger private hospitals with more resources for quality improvement [156]. In this sense, if the goal is to maximize quality improvements and cost savings, then LH interventions or similar methodologies (e.g., Virginia Mason Production System) must occur institution-wide, i.e., in both ambulatory care and inpatient settings [45]. We found one intervention using lean-six sigma and BPPI [19] and other using lean and RIE [8] as examples of others combinations of lean and complementary tools. BPPI is a multi-disciplinary, institutional, performance improvement initiative with the goal of decreasing ED walkouts and boarding hours, inpatient LOS and increasing the number of patients with written discharge orders before noon. On the other hand, the so called Kaizen blitz, or rapid improvement event (RIE), is a focused, fast performing and significant changes initiating activity used for general modification and redesign of observed processes and identified problems [157]. Both BPPI and RIE might differ in scope, but complement lean interventions by improving patient flow and efficiency outcomes. Similarly, other studies have shown that multimodal interventions result in improved outcomes [158,159,160].

Most studies used the value stream map tool to represent both the current and future states of patient flow, thereby confirming the great importance and usefulness to identify VA and NVA activities. This importance has been pointed out by other studies [134,161,162,163,164]. Other common tools include standard work, i.e., a concept whereby each work activity is precisely described with a specific cycle time, task sequence, and other steps involved within the process [95], and is considered a prerequisite for flow [10]; the 5’S program, which is used to eliminate clutter [165] and enhance the standardization of stock, as well as to systematically organize the unit and streamline the documentation processes [97]; cause and effect analyses, which are used to map the possible causes of a problem into categories [90,166]; and Kaizen, which is a philosophy of continuous incremental improvement over time and space [29,99,104]. These findings are similar with those reported in previous studies [44,62,161], and sustain the assertion that most LH interventions focus more on tools related to assessment and improvement and less on processes-monitoring tools.

Even though lean theory assumes a holistic view [62,167], most interventions occurred in a particular process or department, rather than in the whole organization. According to our results, OR accounts for the area with the largest amount of LH interventions (21), while none were implemented in a whole healthcare organization. This is consistent with the results from [65]. Therefore, small and focalized improvements support organizations sustain momentum, and any early achievement is vital to keep people from becoming dispirited [27]. Organizations with more experience could perform larger and longer projects.

The time frame of most studies was longer than one year (32 out of 39). However, only around 15% of all studies conducted a follow-up process longer than one year. These results hinder to confirm the sustainability of the achievements, and may be related to the “project fatigue” in hospitals since so many difficulties within their facilities need attention [168]. Hereafter, a brief follow-up analysis might not be an appropriate indicator of improvement. Some other characteristics that might compromise the sustainability of LH achievements include increased patient volume [138], a poor understanding of the organizational context [169], insufficient space and time for coherent team co-operative improvement, the tension between promoting staff ownership and providing direction [88], the incomplete or slow adoption of the interventions [93], and a lack of standardization [48]. Naturally, to fully realize the potential benefits of LH, organizations need to minimize the impact of such barriers and capitalize on facilitating conditions that are specific to their local contexts [134]. Once institutional rules and dogma are changed, culture and workflow improve [103].

The successful implementation of lean or any other improvement framework requires that the hospital and medical leadership all be strong supporters of the methodology, speak the same process improvement language, and are able to generate support and resources [10]. Besides, when LH is properly executed and is owned by the frontline workers, it can yield improvements in care metrics [9] because the employees are trained to become project leaders for improvement [170]. Engagement and empowerment alone, however, will not drive or sustain such improvement. They must be combined with a new breed of leadership that focuses on patient outcomes and performance measurements as a key motivator [171], along with effective communication and team work [118]. Here, senior leadership plays a critical role [25] by developing supporting structures such as visual control, goal deployment, short daily meetings, two-way communication flow, and a system of continuous improvement [172]. Additionally, the contributions from team members with different perspectives and disciplines was noted in most of the reviewed studies, showing better performance in patient flow indicators. Thus, the team—whether multidisciplinary teams [17,21,100], interprofessional teams [27], work stream teams [22], value teams [20], project teams [91,98], or Kaizen teams [99]—is vital in getting ‘‘buy in’’ from all the stakeholders involved [27], principally because LH continues to sustain a multidisciplinary problem-solving perspective, as demonstrated by the joint ownership of performance measures [1].

Healthcare organizations are currently subject to compliance with standards, targets, and benchmarks, which serve as a reference of minimum performance levels for safety and patient flow [144]. However, the timeframes and metrics for patient throughput differ widely in both practice and literature [120]. For example, the average LOS in hospitals for acute care among OECD countries is 6.5 days, with Turkey (4.1 days) being the shortest and Japan the longest (16.2 days) [173]. In this research, the average LOS before LH was 22.9 days, and 12.5 days after the intervention, but these values depend on many factors, such as patient variables [174], treatments, and settings; e.g., after LH, the LOS in the rehabilitation ward was 58.3 days [85], 22 days in the ICU [17], and 5.3 days in the trauma center [95]. Despite the contexts for standards or target compliance, we found that few studies discussing meeting local or national standards [47,87], national rates [90], organizational goals [3], targets [8], internal benchmarks [89], or consortium benchmarks [28]. Instead, the specified goal was commonly to improve performance.

### 4.7. Risk of Bias

In terms of risk of bias, 72% of the interventions were assessed as moderate and the rest as serious. None were evaluated as critical or as low risk since only exceptionally will a non-randomized study of interventions (NRSI) be assessed as at low risk of bias due to confounding [82,83]. Our risk of bias analysis is consistent with [82], which anticipated that most NRSI will be judged as at least at moderate overall risk of bias. The relative high number of studies with moderate or serious risk of bias might be debatable; however, it signifies our decision to include all those studies meeting the criteria for inclusion and provide a general viewpoint of the LH phenomenon, as recommended when risks of bias vary across studies [175]. Regarding the domains of bias, all studies were evaluated as serious in the bias due to selection of participants. Selection bias occurs when some eligible participants, or some follow-up time of some participants, or some outcome events, are excluded in a way that leads to the association between intervention and outcome in the NRSI differing from the association that would have been observed in the target trial [84]. In this research, none of the selected studies involved RCTs; moreover, the selection of participants into most of the studies was related to intervention and outcome. Finally, the lack of data prevented us from adjusting these types of bias as indicated by [82].

## 5. Limitations

Our study has several major limitations. Firstly, differences in data (patient volume, settings, and data gathering/processing approaches) and the multi-component nature of LH, limit us to generalize the results. Secondly, studies’ heterogeneity and the risk of bias prevented us from carrying out a meta-analysis to determine causal relationships. Thirdly, the majority of the studies were observational pre–post designs. Thus, the lack of randomization, the lack of matched comparison groups, and the potential existence of confounding variables limited the outcome improvements from being causally related to the LH interventions. Moreover, the lack of reliable measures of confounding domains led to the bias due to confounding. Both baseline confounding and time-varying confounding were common in NRSI, and along with bias due to selection of participants, might represent a limitation when estimating the true effect. Finally, there is a possibility that the “Hawthorne effect” led to the improvements reported in the studies, even though the changes in outcomes, as demonstrated in the statistical tests, suggest that the results were more probable due to the LH intervention.

## 6. Conclusions

On the basis of our findings, we have summarized the main results from the LH interventions within inpatient care. As stated by most authors, LH guided and facilitated the identification of non-value activities in their processes, thereby facilitating actions to reduce them, while improving the efficiency of service. According to our findings, excessive LOS is critical for both patient safety and hospital costs, hence, delays in some procedures might lead to an extended stay and thus increase discomfort among hospitalized patients and compromise the capacity of beds. To achieve this goal, an efficient perioperative process should have a high OTS rate since delays and cancellations lead to underutilized facilities and dissatisfaction among personnel.

Bearing in mind the dimensions of quality of care [176], according to our evidence, LH contributes to the provision of efficient and accessible service through a reduction in the length of stay and outcomes associated to the length in time of activities related to the perioperative process and inpatient care, such as TOT, TAT, OTS, boarding time, and discharge time. Moreover, our findings suggest that LH does not contribute to the changes in readmission rates and highlight the important relationship between capacity and demand. By reducing the time-length of the outcomes reported, healthcare professionals increased their capacity, which is crucial to improving the flow of patients to meet demands. In this regard, LH is an important support and, by using a complementary tool (such as six-sigma) that focuses on variation reduction, might help level patient flow and solve more complicated problems, as long as the organization provides support. Likewise, if properly supported, LH might contribute healthcare organizations comply with targets and standards associated to timely and effective care (throughput).

Notwithstanding the improvement in outcomes related to efficiency and patient flow, indication of the LH effect on patient/staff satisfaction is still scarce among studies; similarly, although more studies are translating the obtained achievements of LH into savings, there is still a gap to fill.

## 7. Future Research

We suggest considering variables that might affect inpatient processes such as economic, cultural, and regional characteristics. Additionally, relevant tools and techniques and critical success factors in the implementation of LH within inpatient care should be evaluated. Despite the largely positive findings of LH intervention, caution should be taken in generalizing such findings. Consequently, additional research involving both high quality observational studies and randomized controlled trials is also recommended.

## Figures and Tables

**Table 1:** Direction of Findings per Main Outcomes.

Author	LOS			TOT			TAT			On-Time Starts			Discharge Order Time			Boarding Time			Readmission		
	-	NC	+	-	NC	+	-	NC	+	-	NC	+	-	NC	+	-	NC	+	-	NC	+
Iannettoni, 2011	√																				
Hseng-Long, 2011	√																				
Gayed, 2013	√																				
Dela Lama, 2013	√																				
Beck, 2016	√															√					
Castaldi, 2016				√								√									
Trzeciak, 2018	√																				
Burkitt, 2009		√																			
New, 2016		√																		√	
Collar, 2012				√			√														
Artenstein, 2017	√												√			√					
Hassanain, 2016					√							√									
Yousri, 2011		√																			
Montella, 2017	√																				
Cima, 2011				√								√									
Singh, 2014							√														
Bender, 2015					√							√									
Beck, 2015		√											√								
Tagge, 2017				√			√														
Toledo, 2013	√																			√	
Fairbanks, 2007							√					√									
Molla, 2018		√											√							√	
Niemeijer, 2010	√																			√	
Sayeed, 2018	√																			√	
Brunsman, 2018	√																				
Johnson, 2016		√																	√		
Sirvent, 2016		√											√			√				√	
Vose, 2014																√					
Niemeijer, 2013	√																				
Sorensen, 2019	√												√						√		
Moo-Young, 2019	√													√						√	
Cerfolio, 2019				√																	
Ankrum, 2019				√																	
Peter, 2011												√									
Allen, 2009													√								
El-Eid, 2015	√												√								
Vijay, 2014													√								
Deldar, 2017												√									
Adams, 2004							√														
Total	16	7	N	6	2	N	5	N	N	N	N	7	8	1	N	4	N	N	2	7	N

**Note.** A mark check indicates interventions reporting an outcome. For the outcome direction, (-) denotes that an outcome decreased, NC means no change in outcome, and (+) means the outcome increased. LOS indicates length of stay; TOT, turnover time; TAT, turnaround time; N, no report. The last name of the main author and the publication year are shown.

**Table 2:** Main Outcomes of Lean Healthcare Intervention.

First Author, Year, Country	Setting, Study Design, (n), Time Frame	Main Intervention	Outcomes	Summary of Findings
Iannettoni, 2011, USA [99]	Cardiothoracic, Pre–Post, (n = 64), 60 mo.	Lean and Kaizen	Length of stay (Average)	Decreased from 14 days to 5 days
Hseng-Long, 2011, Taiwan [47]	Cardiology, Pre–Post, (n = 46), 15 mo.	Lean and Six Sigma	Length of stay (Average)	Decreased by 3 days
Gayed, 2013, USA [48]	Department of Surgery, Pre–Post, (n = 540), 35 mo.	Lean Six Sigma	Length of stay (Mean)	Decreased from 5.3 days to 3.4 days (*p* < 0.001)
De la Lama, 2013, Spain [85]	Rehabilitation ward, Pre–Post, (n = 75,490), 15 mo.	Six Sigma	Length of stay (Mean)	Decreased from 164.1 days to 58.2 days (*p* < 0.001)
Beck, 2016, USA [3]	Emergency department, Pre–Post, (n = 6906), 25 mo.	Lean	Discharge order entry time (Median)	Decreased from 1:43 pm to 11:28 am (*p* < 0.0001)
			Discharge time (Median)	Decreased from 3:25 pm to 2:25 pm (*p* < 0.0001)
			Discharge before noon (Percentage)	Increased from 14% to 26% (*p* < 0.0001)
			Boarding time (Median)	Decreased from 176 min to 127 min (*p* < 0.0001)
			Length of stay (Average)	Decreased from 3.8 days to 3.4 days
Castaldi, 2016, USA [8]	Operating room, Pre–Post, 32 mo.	Lean and RIE	OR turnover time (Average)	Decreased from 54 min to 41 min (*p* = 0.0001)
			On-time Starts (Percentage)	Increased from 54% to 84% (*p* = 0.0001)
Trzeciak, 2018, USA [17]	Intensive care unit, Cohort study, (n = 269), 24 mo.	Lean Six Sigma	Length of stay (Median)	Decreased from 29 days to 22 days (*p* < 0.001)
Burkitt, 2009, USA [86]	Department of Surgery, Cohort study, (n = 1779), 48 mo.	TPS	Length of stay (Median)	Non-significant change (*p* = 0.90)
New, 2016, UK [88]	Orthopedic trauma theatre, Pre–Post, (n = 1041), 18 mo.	Lean	Length of stay (Mean)	Non-significant change (*p* = 0.396)
			Readmission (Proportion)	Non-significant change (*p* = 0.30)
Collar, 2012, USA [21]	Operating room, Cohort study, (n = 199), 18 mo.	Lean	Turnover time (Mean)	Decreased from 38.4 min to 29 min (*p* < 0.001)
			Turnaround time (Mean)	Decreased from 89.5 min to 69.3 min (*p* < 0.001)
Artenstein, 2017, USA [19]	Emergency Department, Pre–Post, 24 mo.	Lean Six Sigma and BPPI	Length of stay (Mean)	Decreased from 5.3 days to 5 days (*p* < 0.005)
			Boarding time (Mean)	Decreased from 7.6 h to 5.5 h (*p* = 0.007)
			Discharge before noon (Percentage)	Increased from 43% to 54.1% (*p* < 0.001)
Hassanain, 2016, Saudi Arabia [26]	Operating room, Cohort study, 28 mo.	Lean	On-time start (Percentage)	Increased from 14% to 34% (*p* < 0.001)
			Room turnover time (Median)	Non-significant change
Yousri, 2011, UK [87]	Department of Surgery, Pre–Post, (n = 608), 24 mo.	Lean	Length of stay (Median)	Non-significant change (*p* = 0.178)
Montella, 2017, Italy [100]	Department of Surgery, Pre–Post, (n = 22,262), 48 mo.	Lean Six Sigma	Length of stay (Mean)	Decreased from 45 days to 36 days (*p* = 0.038)
Cima, 2011, USA [22]	Operating room, Pre–Post, (n = 8497), 18 mo.	Lean Six Sigma	On-time starts (Percentage)	TS increased from 50% to 80% (*p* < 0.05); GYN increased from 64% to 92% (*p* < 0.05); Gen/CRS increased from 60% to 92% (*p* < 0.05)
			Turnover time (Average)	TS decreased from 40 min to 30 min (*p* < 0.05); GYN decreased from 35 min to 20 min (*p* < 0.05); Gen/CRS decreased from 34 min to 23 min (*p* < 0.05)
Singh, 2014, India [23]	Operating room, Pre–Post, (n = 231), 6 mo.	Lean Six Sigma	Turnaround time (Mean)	Decreased from 17.6 min to 10.4 min (*p* < 0.0002)
Bender, 2015, USA [27]	Operating room, Pre–Post, (n = 25,903), 36 mo.	Lean Six Sigma	On-time starts (Percentage)	Increased from 32% to 73%
			Turnover time (Average)	Non-significant change
Beck, 2015, USA [89]	Inpatient pediatric service, Pre–Post, (n = 3509), 12 mo.	Lean Six Sigma	Time of patient discharge (Median)	Decreased from 15:48 min to 14:15 min (*p* < 0.0001)
			Patients discharged by noon (Proportion)	Decreased from 27% to 14% (*p* < 0.0001)
			Length of stay (Mean)	Non-significant change (*p* = 0.864)
Tagge, 2017, USA [24]	Operating room, Pre–Post, (n = 612), 6 mo.	Lean Six Sigma	Turnover time (Median)	Decreased from 41 min to 32 min (*p* < 0.0001)
			Turnaround time (Median)	Decreased from 81.5 min to 71 min (*p* < 0.0001)
Toledo, 2013, USA [28]	Organ transplant center, Pre–Post, (n = 103), 48 mo.	Lean Six Sigma	Length of stay (Median)	Decreased from 11 days to 8 days (*p* < 0.05)
			30-day readmission (Rate)	Non-significant change (*p* = 0.63)
Fairbanks, 2007, USA [101]	Operation Room, Pre–Post, 12 mo.	Lean Six Sigma	On-time start (Percentage)	Increased from 12% to 89%
			Turnaround time (Mean)	Decreased from 23.8 min to 17.9 min
Molla, 2018, USA [20]	Operating room, Pre–Post, (n = 1471), 28 mo.	Lean Six Sigma	Discharge orders released by 10:00 (Percentage)	Increased by 21.3% (*p* < 0.001)
			Patients discharged by noon (Percentage)	Increased by 7.5% (*p* = 0.001)
			30-day readmission (Rate)	Non-significant change (*p* = 0.492)
			Length of stay (Mean)	Non-significant change (*p* = 0.153)
Niemeijer, 2010, The Netherlands [98]	Trauma Care, Pre–Post, (n = 1693), 18 mo.	Lean Six Sigma	Length of stay (Average)	Decreased from 11.8 days to 8.5 days
Sayeed, 2018, USA [95]	Operating room, Pre–Post, (n = 505), 24 mo.	Lean Six Sigma	Length of stay (Average)	Decreased from 6 days to 5.2 days (*p* = 0.02)
			30-day readmissions (Rate)	Non-significant change (*p* = 0.13)
Brunsman, 2018, USA [18]	Inpatient pharmacy, Cohort study, (n = 102), 15 mo.	Lean	Length of stay (Median)	Decreased from 22.9 days to 13.2 days (*p* = 0.049)
Johnson, 2016, USA [90]	Emergency department, Pre–Post, (n = 1394), 24 mo.	Lean Six Sigma	Heart failure patient’s readmission (Average)	Decreased from 28.4% to 18.9% (*p* < 0.01)
			Length of stay (Mean)	Non-significant change (*p* = 0.70)
Sirvent, 2016, Spain [29]	Intensive care unit, Pre–Post, (n = 1388), 12 mo.	Lean	ICU boarding time (Mean)	Decreased from 360.8 min to 276.7 min (*p* = 0.036)
			Length of stay in ICU (Mean)	Non-significant change (*p* = 0.992)
			Readmissions (Percentage)	Non-significant change (*p* = 0.966)
Vose, 2014, USA [91]	Emergency department, Pre–Post, 24 mo.	Lean	Boarding time (Average)	Decreased from 58.9 min to 43.6 min
Niemeijer, 2013, The Netherlands [102]	Department of Surgery, Pre–Post, (n = 332), 45 mo.	Lean Six Sigma	Length of stay (Average)	Decreased from 13.5 days to 9.3 days (*p* = 0.000)
Sorensen, 2019, USA [92]	Department of Surgery, Pre–Post, (n = 4253), 36 mo.	Lean	Length of stay (Mean)	Decreased from 3.2 days to 2.4 days (*p* < 0.001)
			30-day readmission (Percentage)	Decreased from 3.1% to 1.1% (*p* = 0.032)
			Discharge to home (vs. rehabilitation facility or skilled nursing facility) (Percentage)	Increased from 72% to 91% (*p* < 0.001) for hip patients; Increased from 70% to 87% (*p*< 0.001) for knee patients
Moo-Young, 2019, USA [93]	Pediatric gastroenterology, Pre–Post, (n = 355), 12 mo.	Lean Six Sigma	30-day readmission (Rate)	Non-significant change (*p* = 0.54)
			Discharged before 1 pm (Percentage)	Non-significant change
			Length of stay (Mean)	Decreased from 5.7 days to 4.7 days (*p* = 0.055)
Cerfolio, 2019, USA [103]	Operating room, Pre–Post, (n = 128), 6 mo.	Lean	OR turnover time (Median)	Decreased from 37 min to 14 min (*p* < 0.0001)
Ankrum, 2019, USA [104]	Isolation room, Pre–Post, (n = 38), 2 mo.	Lean	Room turnover time (Median)	Decreased from 130 min to 65 min (*p* < 0.0001)
Peter, 2011, USA [105]	Operating room, Pre–Post, 24 mo.	Lean Six Sigma	Cases starting on time (Percentage)	Increased from 13% to 80%
Allen, 2009, USA [106]	Hospital discharge process, Pre–Post, (n = 150), 6 mo.	Six Sigma	Discharge time (Average)	Decrease from 3.3 h to 2.8 h (*p* = 0.068)
El-Eid, 2015, Lebanon [107]	Emergency department, Pre–Post, (n = 17,054), 10 mo.	Six Sigma	Discharge time (Mean)	Decreased from 2.2 h to 1.7 h. (*p* < 0.001)
			Length of stay (Mean)	Decreased from 3.4 days to 3.1 days (*p* < 0.001)
Vijay, 2014, India [108]	Department of Surgery, Pre–Post, (n = 120), 3 mo.	Six Sigma	Cycle time of patient discharge process (Average)	Decreased from 234 min to 143 min
Deldar, 2017, USA [109]	Operating room, Pre–Post, (n = 4492), 7 months	Lean	On-time starts (Percentage)	Increased from 57% to 69% (*p* < 0.01)
Adams, 2004, USA [110]	Operating room, Pre–Post, (n = 96), 8 mo.	Six Sigma	Turnaround time between cases in the OR (Mean)	Decreased from 22.8 min to 15.6 min

**Note.** OR indicates operating room; RIE, Rapid improvement event; ED, Emergency department; TPS, Toyota Production System; BPPI, Baystate Patient Progress Initiative; mo. Months; h, Hours; min, Minutes; TS, Thoracic surgery; GYN, Gynecologic oncology surgery; Gen/CRS, General and colorectal surgery. The last name of the main author and the publication year are shown.

## Supplementary Materials

The following are available online at https://www.mdpi.com/1660-4601/17/15/5609/s1, Table S1: PRISMA checklist, Data S1: Search strategy, Table S2: Geographical distribution of studies selected, Table S3: Distribution per year of studies selected, Table S4: Summary of findings of LH, and Table S5: Risk of bias.
