# Supplementary material for: Assessing the Impact of Lean Healthcare on Inpatient Care: A Systematic Review
Source: Int J Environ Res Public Health. 2020 Aug 4;17(15):5609. doi: 10.3390/ijerph17155609 (PMC7432925; doi:10.3390/ijerph17155609)
Supplement: Supplementary file 1 [file ijerph-17-05609-s001.pdf]

Table 1. PRISMA Checklist.

Section/topic	#	Checklist item	Reported on section
**TITLE**			
Title	1	Identify the report as a systematic review, meta-analysis, or both.	Title
**ABSTRACT**			
Structured summary	2	Provide a structured summary including, as applicable: background; objectives; data sources; study eligibility criteria, participants, and interventions; study appraisal and synthesis methods; results; limitations; conclusions and implications of key findings; systematic review registration number.	Abstract
**INTRODUCTION**			
Rationale	3	Describe the rationale for the review in the context of what is already known.	Introduction
Objectives	4	Provide an explicit statement of questions being addressed with reference to participants, interventions, comparisons, outcomes, and study design (PICOS).	Introduction
**METHODS**			
Protocol and registration	5	Indicate if a review protocol exists, if and where it can be accessed (e.g., Web address), and, if available, provide registration information including registration number.	Methods
Eligibility criteria	6	Specify study characteristics (e.g., PICOS, length of follow-up) and report characteristics (e.g., years considered, language, publication status) used as criteria for eligibility, giving rationale.	Methods
Information sources	7	Describe all information sources (e.g., databases with dates of coverage, contact with study authors to identify additional studies) in the search and date last searched.	Methods
Search	8	Present full electronic search strategy for at least one database, including any limits used, such that it could be repeated.	Data S1
Study selection	9	State the process for selecting studies (i.e., screening, eligibility, included in systematic review, and, if applicable, included in the meta-analysis).	Methods
Data collection process	10	Describe method of data extraction from reports (e.g., piloted forms, independently, in duplicate) and any processes for obtaining and confirming data from investigators.	Methods
Data items	11	List and define all variables for which data were sought (e.g., PICOS, funding sources) and any assumptions and simplifications made.	Methods
Risk of bias in individual studies	12	Describe methods used for assessing risk of bias of individual studies (including specification of whether this was done at the study or outcome level), and how this information is to be used in any data synthesis.	Methods

Summary measures	13	State the principal summary measures (e.g., risk ratio, difference in means).	Methods
Synthesis of results	14	Describe the methods of handling data and combining results of studies, if done, including measures of consistency (e.g., I^2) for each meta-analysis.	N/A
**Section/topic**	**#**	**Checklist item**	**Reported on page #**
Risk of bias across studies	15	Specify any assessment of risk of bias that may affect the cumulative evidence (e.g., publication bias, selective reporting within studies).	N/A
Additional analyses	16	Describe methods of additional analyses (e.g., sensitivity or subgroup analyses, meta-regression), if done, indicating which were pre-specified.	N/A
**RESULTS**			
Study selection	17	Give numbers of studies screened, assessed for eligibility, and included in the review, with reasons for exclusions at each stage, ideally with a flow diagram.	Results and Figure 1
Study characteristics	18	For each study, present characteristics for which data were extracted (e.g., study size, PICOS, follow-up period) and provide the citations.	Table 2 and Table S4
Risk of bias within studies	19	Present data on risk of bias of each study and, if available, any outcome level assessment (see item 12).	Table S5
Results of individual studies	20	For all outcomes considered (benefits or harms), present, for each study: (a) simple summary data for each intervention group (b) effect estimates and confidence intervals, ideally with a forest plot.	Table 2 and Table S4
Synthesis of results	21	Present results of each meta-analysis done, including confidence intervals and measures of consistency.	N/A
Risk of bias across studies	22	Present results of any assessment of risk of bias across studies (see Item 15).	N/A
Additional analysis	23	Give results of additional analyses, if done (e.g., sensitivity or subgroup analyses, meta-regression [see Item 16]).	N/A
**DISCUSSION**			
Summary of evidence	24	Summarize the main findings including the strength of evidence for each main outcome; consider their relevance to key groups (e.g., healthcare providers, users, and policy makers).	Results
Limitations	25	Discuss limitations at study and outcome level (e.g., risk of bias), and at review-level (e.g., incomplete retrieval of identified research, reporting bias).	Discussion
Conclusions	26	Provide a general interpretation of the results in the context of other evidence, and implications for future research.	Conclusions

**FUNDING**			
Funding	27	Describe sources of funding for the systematic review and other support (e.g., supply of data); role of funders for the systematic review.	N/A

Data S1. Search Strategy

Pubmed-Medline

Healthcare units and inpatient care

1. ("Hospital Departments/methods"[Mesh] OR "Hospital Departments/organization and administration"[tiab] OR “Hospital Departments/standards"[tiab] OR “Hospital Departments/statistics and numerical data"[tiab] AND lean healthcare).
2. Lean healthcare AND (admitting OR clinic? OR emergency department? OR emergency medicine OR emergency room? OR emergency service? OR family practice? OR general practice? OR healthcare OR hospital? OR inpatient? OR intensive care OR ICU OR oncology OR outpatient? OR pharmacist? OR readmission? OR trauma center? OR trauma service? OR trauma care OR inpatient care OR primary care OR secondary care OR tertiary care).

Lean

1. (“Lean healthcare”[tiab] OR “lean thinking”[tiab] OR “lean manufacturing”[tiab] OR (lean[tiab] AND sigma[tiab]) OR toyota[tiab] OR “lean principles”[tiab] OR “lean management”[tiab] OR lean process[tiab] OR lean process management[tiab] OR lean healthcare approach[tiab]).
2. Lean healthcare AND (approach OR business model? OR care OR collaborate* OR design* OR healthcare OR implementation? OR industry OR initiative? OR intervention* OR leader* OR management OR methodology* OR method? OR organi?ation* OR planning OR philosophy OR practice* OR principle* OR process improvement? OR production OR program? OR quality OR redesign* OR reengineer* OR restructure* OR reorgani* OR safety OR sigma OR strategy OR thinking OR tool).

Inpatient care outcomes

1. ((("Length of Stay/organization and administration"[Mesh] OR "Length of Stay/standards"[Mesh] OR "Length of Stay/statistics and numerical data"[Mesh])) OR "Patient Outcome Assessment/organization and administration"[Mesh]) AND ("Total Quality Management/methods"[Mesh] OR "Total Quality Management/organization and administration"[Mesh] OR "Total Quality Management/statistics and numerical data"[Mesh]).

TAT, TOT, Boarding Time, Discharge, Readmission and On-time starts outcomes

1. ((((((((Turnover time) OR Turnaround time) OR Boarding time) OR discharge order) OR readmission) OR on time starts) AND Lean healthcare) NOT specimens) NOT samples) AND six sigma) AND lean.

The Cochrane Library

Healthcare units and inpatient care

1. Lean healthcare AND (admitting OR clinic? OR emergency department? OR emergency medicine OR emergency room? OR emergency service? OR family practice? OR general practice? OR healthcare OR hospital? OR inpatient? OR intensive care OR ICU OR oncology OR outpatient? OR pharmacist? OR readmission? OR trauma center? OR trauma service? OR trauma care OR inpatient care OR primary care OR secondary care OR tertiary care).

Lean

1. Lean healthcare AND (approach OR business model? OR care OR collaborate* OR design* OR healthcare OR implementation? OR industry OR initiative? OR intervention* OR leader* OR management OR methodology* OR method? OR organi?ation* OR planning OR philosophy OR practice* OR principle* OR process improvement? OR production OR program? OR quality OR redesign* OR reengineer* OR restructure* OR reorgani* OR safety OR sigma OR strategy OR thinking OR tool).

Inpatient care outcomes

1. (Lean and waste). ti,ab. OR (lean adj3 waste). ti,ab.
2. ((Wait$ time? OR reduc$ wait$) and lean). ti,ab. OR ((wait$ time? OR reduc$ wait$) adj4 lean). ti,ab.
3. (Lean and (overcrowd$ OR patient$ flow? OR wait time?)). ti,ab.

TAT, TOT, Boarding Time, Discharge, Readmission and On-time starts outcomes

1. (Turnover time OR Turnaround time OR Boarding time OR discharge order OR readmission OR on time starts) AND (Lean healthcare OR six sigma OR Lean).

EBSCO

Healthcare units and inpatient care

1. ("Hospital Departments/methods"[Mesh] OR "Lean Hospital Departments/organization and administration"[tiab] OR "Lean Hospital Departments/standards"[tiab] OR "Lean Hospital Departments/statistics and numerical data"[tiab] AND lean healthcare).

Lean

1. (Lean healthcare[tiab] OR lean thinking[tiab] OR lean process[tiab] OR lean process management[tiab] OR lean healthcare approach[tiab] OR continuous quality management OR lean six sigma[tiab] OR lean management[tiab] OR lean operations management[tiab] OR total quality management [mesh] AND lean healthcare).

Inpatient care outcomes

1. Lean healthcare AND (Patient flow OR waiting time OR length of stay).

TAT, TOT, Boarding Time, Discharge, Readmission and On-time starts outcomes

1. (Turnover time OR Turnaround time OR Boarding time OR discharge order OR readmission OR on time starts) AND (Lean healthcare OR six sigma OR Lean).

CINAHL

1. *Healthcare units and inpatient care*
2. Lean healthcare AND (surgical OR surgery OR readmission? OR intensive care OR inpatient? OR ICU OR hospitali#ed OR general practice? OR admitting OR clinics OR emergency department? OR emergency room? OR emergency service? OR family practice? OR primary care OR pharmacy OR hospital? OR oncology OR trauma center? OR trauma service?).

Lean

1. Lean healthcare AND (implementation? OR healthcare OR industry OR initiative? OR intervention* OR leader* OR management OR method? OR methodolog* OR planning OR tools OR workshop* thinking OR strategies OR sigma OR quality OR production OR process improvement? OR principles OR principle OR practices OR practice OR philosophy).

Inpatient care outcomes

1. (TI ((wait* time? OR reduc* wait*) and lean) OR AB (lean (overcrowd* OR patient* flow?))) OR ((wait* time? OR reduc* wait*) lean)) OR (TI (lean and (overcrowd* OR patient* flow?)).

TAT, TOT, Boarding Time, Discharge, Readmission and On-time starts outcomes

1. (Turnover time OR Turnaround time OR Boarding time OR discharge order OR readmission OR on time starts) AND (Lean healthcare OR six sigma OR Lean).

Web of science

Healthcare Units and inpatient care

1. Lean healthcare AND (healthcare units OR intensive care unit OR clinical management OR health facilities health center OR patient centered care OR patient and family centered care OR public healthcare management).

Lean

1. Lean healthcare AND (management OR six sigma OR thinking OR process OR management OR operations OR lean process management OR lean healthcare approach OR lean six sigma OR total quality management).

Inpatient care outcomes

1. Lean healthcare AND (length of stay OR wait time OR length in hospital OR patient satisfaction OR wait times and length of hospitalization OR patient flow length of stay prediction OR length of stay cost OR hospital stays OR stay length OR decrease hospital length of stay OR wait and time OR delay time AND lean healthcare).

TAT, TOT, Boarding Time, Discharge, Readmission and On-time starts outcomes

1. (Turnover time OR Turnaround time OR Boarding time OR discharge order OR readmission OR on time starts) AND (Lean healthcare OR six sigma OR Lean)

Scopus

Healthcare Units and Inpatient care

1. Healthcare units OR intensive care unit OR clinical management OR health facilities health center OR patient centered care OR patient and family centered care OR public healthcare management.

Lean

1. (Lean management OR lean six sigma OR lean thinking OR lean process OR lean principles OR lean operations and total quality management OR continuous quality improvement and lean methodology OR lean process management OR lean implementation).

Inpatient care outcomes

1. Lean healthcare AND (Patient flow OR waiting time OR length of stay).

TAT, TOT, Boarding Time, Discharge, Readmission and On-time starts outcomes

1. (Turnover time OR Turnaround time OR Boarding time OR discharge order OR readmission OR on time starts) AND (Lean healthcare OR six sigma OR Lean).

Table S2. Geographical distribution of studies selected.

Country	Total
**USA**	27
**Taiwan**	1
**Spain**	2
**Netherlands**	2
**UK**	2
**Saudi Arabia**	1
**Italy**	1
**India**	2
**Lebanon**	1

Table S3. Distribution per year of studies selected

Year	Total
**2011**	5
**2016**	5
**2013**	4
**2014**	4
**2017**	4
**2018**	4
**2019**	4
**2015**	3
**2009**	2
**2004**	1
**2007**	1
**2010**	1
**2012**	1

Table 4. Summary of Findings of Lean Healthcare Intervention.

First Author, Year, Country	Aim of Study	Setting, Study Design, (n), Time Frame	Main Intervention	Outcomes	Summary of findings
Iannettoni, 2011, USA [1]	To improve patient outcomes, increasing patient satisfaction, and creating efficiencies in the system	Cardiothoracic, Pre-Post, (n=64), 60 months	Lean and Kaizen	Cost per case<br>Length of stay (Average)<br>CEGA leak (Rate)<br>Savings<br>Operative time	Cost reduction of 43%<br>Decreased from 14 to 5 days<br>Decreased from 12% to zero leaks<br>Daily savings of $4,500<br>Decreased from 348 to 189 min
Hseng-Long, 2011, Taiwan [2]	To improve the medical process of acute myocardial infarction	Cardiology, Pre-Post, (n=46), 15 months	Lean and Six Sigma	Wait time to see a doctor (Mean)<br>Process cycle efficiency<br>Length of stay (Average)<br>Saving in medical resource	Decreased from 139.2 to 57.9 min<br>Increased from 32.2 to 51.8%<br>Decreased by 3 days<br>Estimated savings of NT $4.422 million
Gayed, 2013, USA [3]	To determine the effectiveness of Lean Six Sigma process improvement methods	Department of Surgery, Pre-Post, (n=540), 35 months	Lean Six Sigma	Length of stay (Mean)<br>Return on investment	Decreased from 5.3 to 3.4 days ($p<0.001$)<br>Estimates of $1 million return annually
De la Lama, 2013, Spain [4]	To improve internal processes in a hospital center through three pilot projects	Rehabilitation ward, Pre-Post, (n=75,490), 15 months	Six Sigma	Delay in outpatients (Mean)<br>Absenteeism (Percentage)<br>Length of stay (Mean)	Decreased from 523.5 to 125.2 min ($p<0.001$)<br>Decreased from 11.4 to 6.2% (NSS)<br>Decreased from 164.1 to 58.2 days ($p<0.001$)
Beck, 2016, USA [5]	To improve emergency department throughput and reduced emergency department boarding	Emergency department, Pre-Post, (n=6,906), 25 months	Lean	Discharge order entry time (Median)<br>Discharge time (Median)<br>Patients discharged before noon (Percent)<br>ED boarding time (Median)	Decreased from 1:43 pm to 11:28 am ($p<0.0001$)<br>Decreased from 3:25 to 2:25 pm ($p<0.0001$)<br>Increased from 14 to 26% ($p<0.0001$)

				Length of stay (Average)	Decreased from 176 to 127 min (p<0.0001)<br>Decreased from 3.8 to 3.4 days
Castaldi, 2016, USA [6]	To increase efficiency in the entire peri-operative process and increase operating room utilization	Operating room, Pre-Post, 32 months	Lean and RIE	OR turnover time (Average)<br>On-time Starts (Percentage)<br>OR utilization (Percentage)<br>Cancellations on the day of surgery	Decreased from 54 to 41 min (p=0.0001)<br>Increased from 54 to 84% (p=0.0001)<br>Increased from 65.5 to 80% (p=0.0007)<br>No statistically significant change (p=0.69)
Trzeciak, 2018, USA [7]	To reduce hospital LOS and associated costs of care for patients with prolonged mechanical ventilation	Intensive care unit, Cohort study, (n=269), 24 months	Lean Six Sigma	Length of stay (Median)<br>Hospital direct cost per case (Median)	Decreased from 29 to 22 days (p<0.001)<br>Decreased from $66,335 to $48,370 (p<0.001)
Burkitt, 2009, USA [8]	To reduce nosocomial MRSA infections on surgical unit and length of stay	Department of Surgery, Cohort study, (n=1,779), 48 months	TPS	Appropriate perioperative (Proportion)<br>Length of stay (Median)	Increased from 23.4 to 44% (p<0.01)<br>No statistically significant change (p=0.90)
New, 2016, UK [9]	To examine the effectiveness of a "systems" approach using Lean methodology to improve surgical care	Orthopedic trauma theatre, Pre-Post, (n=1,041), 18 months	Lean	Length of stay (Mean)<br>90 Days Readmissions (Proportion)	No statistically significant change (p=0.396)<br>No statistically significant change (p=0.30)
Collar, 2012, USA [10]	To improve efficiency and profitability and preserves team morale and educational opportunities	Operating room, Cohort study, (n=199), 18 months	Lean	Turnover time (Mean)<br>Turnaround time (Mean)<br>Employee satisfaction<br>Annual Opportunity revenue	Decreased from 38.4 to 29 min (p<0.001)<br>Decreased from 89.5 to 69.3 min (p<0.001)<br>Increased from 2.9 to 3.6 (p=0.011)<br>Annual revenue of $330,000
Artenstein, 2017, USA [11]	To optimize patient progress for adult patients	Emergency Department, Pre-Post, 24 months	Lean Six Sigma and BPPI	Length of stay (Mean)<br>ED boarding time (Mean)<br>ED walkout per day (Rate)	Decreased from 5.3 to 5 days (p<0.005)<br>Decreased from 7.6 to 5.5 h (p=0.007)

				Discharge orders before noon (Percentage)<br>Patients seen on daily IPOC rounds (Percent)<br>Inpatient capacity	Decreased from 31 to 21 patients (p=0.01)<br>Increased from 43 to 54.1% (p < 0.001)<br>Increased from 44 to 83% (p<0.001)<br>Increased 20 open beds.
Hassanain, 2016, Saudi Arabia [12]	To improve the utilization of the operating room	Operating room, Cohort study, 28 months	Lean	On-time start (Percentage)<br>OR utilization (Percentage)<br>Room turnover time (Median)	Increased from 14 to 34% (p<0.001)<br>Increased from 39 to 49% (p<0.001)<br>No statistically significant change
Yousri, 2011, UK [13]	To improve the outcome of fracture neck of femur patients	Department of Surgery, Pre-Post, (n=608), 24 months	Lean	30-day mortality (Rate)<br>Overall mortality (Rate)<br>Door to theatre time (≤24 h) (Percentage)<br>Door to theatre time (>48 h) (Percentage)<br>Admission to a trauma ward (Percentage)<br>Length of stay (Median)	Decreased from 11.7 to 6.7% (p=0.034)<br>Decreased from 20.7 to 11.4% (p=0.002)<br>No statistically significant change (p=0.08)<br>No statistically significant change (p=0.481)<br>No statistically significant change (p=0.421)<br>No statistically significant change (p=0.178)
Montella, 2017, Italy [14]	To reduce the number of patients affected by sentinel bacterial infections who are at risk of HAI	Department of Surgery, Pre-Post, (n=22,262), 48 months	Lean Six Sigma	Length of stay (Mean)<br>Associated infections (Percentage)	Decreased from 45 to 36 days (p=0.038)<br>Decreased from 0.3 to 0.2% (p=0.031)

Cima, 2011, USA [15]	To improve Operating Room efficiency	Operating room, Pre-Post, (n=8,497), 18 months	Lean Six Sigma	On-time starts (Percentage)	TS increased from 50 to 80% (p<0.05); GYN increased from 64 to 92% (p<0.05); Gen/CRS increased from 60 to 92% (p<0.05)
				Operations past 5 PM (Percentage)	TS decreased from 34 to 36% (p=0.34); GYN decreased from 42 to 36% (p<0.05); GEN decreased from 37 to 31% (p<0.05)
				Turnover time (Average)	TS decreased from 40 to 30 min (p<0.05); GYN decreased from 35 to 20 min (p<0.05); Gen/CRS decreased from 34 to 23 min (p<0.05).
				Staff overtime (Average)	TS decreased from 109 to 92 min; GYN decreased from 106 to 87 min; GEN decreased from 87 to 41 min
				Daily OR capacity	TS increased from 0 to 0.7 ORs per day; GYN increased from 0 to 0.5 ORs per day; GEN increased from 0 to 0.4 ORs per day.
				Change in operating margin (Percentage)	TS increased from 1 to 1.2%; GYN increased from 1 to 1.1%; GEN increased from 1 to 1.5%
Singh, 2014, India [16]	To increase the efficiency of the operating theater utilization	Operating room, Pre-Post, (n=231), 6 months	Lean Six Sigma	Patient in and Induction begin time (Mean)	Decreased from 5.1 to 3.6 min (p<0.0017)
				Induction End time and Incision (Mean)	Decreased from 15.6 to 12.5 min (p<0.0574)
				Turnaround time (Mean)	Decreased from 17.6 to 10.4 min (p<0.0002)
Bender, 2015, USA [17]	To improve operating room utilization	Operating room, Pre-Post, (n=25,903), 36 months	Lean Six Sigma	Outpatient's readiness on time for surgery	Increased from 59 to 95%<br>Increased from 32 to 73%

				First case on-time starts (Percentage)<br>Block utilization<br>Actual room Utilization<br>Overtime<br>Personnel costs<br>Annual Revenues<br>Turnover time (Average)	Increase from 68 to 74%<br>Increased from 56 to 68%<br>Increased from 7 to 4%<br>Decreased 14 despite 26% more employees<br>Increased more than 10%<br>No statistically significant change
Beck, 2015, USA [18]	To determine the impact of Lean Six Sigma on advancing times of placement of discharge order and patient discharge	Inpatient pediatric service, Pre-Post, (n=3,509), 12 months	Lean Six Sigma	Time of patient discharge (Median)<br>Patients discharged by noon (Proportion)<br>Length of stay (Mean)<br>Patient Satisfaction<br>Revenue	Decreased from 15:48 to 14:15 min (p<0.0001)<br>Decreased from 27 to 14% (p<0.0001)<br>No statistically significant change (p=0.864)<br>Increased from 91 to the 94 percentiles<br>Increased from $275,000 to $412,000
Tagge, 2017, USA [19]	To improving operating room efficiency	Operating room, Pre-Post, (n=612), 6 months	Lean Six Sigma	Turnover time (Median)<br>Turnaround time (Median)	Decreased from 41 to 32 min (p<0.0001)<br>Decreased from 81.5 to 71 min (p<0.0001)
Toledo, 2013, USA [20]	To decrease the length of stay for liver transplant	Organ transplant center, Pre-Post, (n=103), 48 months	Lean Six Sigma	Length of stay after liver transplant (Median)<br>30-day Readmission (Rate)<br>Mortality rates at 30 days and 1 year	Decreased from 11 to 8 days (p<0.05)<br>No statistically significant change (p=0.63)<br>No statistically significant change
Fairbanks, 2007, USA [21]	To improve patient flow in the perioperative environment	Operation Room, Pre-Post, 12 months	Lean Six Sigma	On-time start (Percentage)<br>Turnaround time (Mean)<br>Wait times before surgical procedures<br>Communication of delays<br>Patient perception of how well staff members worked together	Increased from 12 to 89%<br>Decreased from 23.8 to 17.9 min<br>Increased from 85.7 to 88.1 min<br>Increased from 85.9 to 88.2 min<br>Increased from 95.8 to 97.2 (p=0.05)

Molla, 2018, USA [22]	To decrease emergency department throughput time	Operating room, Pre-Post, (n=1,471), 28 months	Lean Six Sigma	Discharge orders released by 10:00 (Percentage)<br>Patients discharged by noon (Percentage)<br>30-day readmission (Rate)<br>Length of stay (Mean)	Increased by 21.3% (p<0.001)<br><br>Increased by 7.5% (p=0.001)<br>No statistically significant change (p=0.492)<br>No statistically significant change (p=0.153)
Niemeijer, 2010, Netherlands [23]	To reduce the mean LOS to create more admission capacity and reduce costs	Trauma Care, Pre-Post, (n=1,693), 18 months	Lean Six Sigma	Length of stay of (Average)<br>Savings<br>Bed availability (Average)<br>Readmission rate	Decreased from 11.8 to 8.5 days<br>Financial benefit of €176,400<br>Increased from 2 to 4.4 beds<br>No change
Sayeed, 2018, USA [24]	To illustrate the application of LSS in the implementation of a hip fracture integrated care pathway	Operating room, Pre-Post, (n=505), 24 months	Lean Six Sigma	Time to surgery (Mean)<br>Patients operated (Percentage)<br>Length of stay (Average)<br>Hospital cost per case<br>30-day readmissions (Rate)<br>Duration of surgery<br>Complication detection<br>Transfusion (Rate)	Decreased from 26.1 to 22.7 h (p=0.06)<br>Decreased from 9.5% to 4.2% (p=0.01)<br>Decreased from 6.0 to 5.2 days (p=0.02)<br>Decreased by 9.7% (p=0.016)<br>No statistically significant change (p=0.13)<br>Decreased from 1.1 to 1.0 h (p=0.03)<br>Increased from 62.4 to 80.1% (p<0.001)<br>Decreased from 58.3 to 50.5% (p=0.07)
Brunsman, 2018, USA [25]	To optimize timely administration of Centers for Medicare and Medicaid Services	Inpatient pharmacy, Cohort study, (n=102), 15 months	Lean	Turnaround time from CMS (Median)<br>Time from order to medication (Median)<br>Savings<br>Time from verification to medication (Median)<br>Length of stay (Median)	Decreased from 120 to 80 min (p=0.014)<br>Increased from 5.5 to 10.5 min (p=0.11)<br>Estimated savings of $250,000<br>Decreased from 116 to 66 min (p=0.005)

					Decreased from 22.9 to 13.2 days (p=0.049)
Johnson, 2016, USA [26]	To investigate the impact on 30-day all-cause readmissions among heart failure patients	Emergency department, Pre-Post, (n=1,394), 24 months	Lean Six Sigma	Heart failure patient's readmission (Average)<br>Length of stay (Mean)<br>Service Savings	Decreased from 28.4 to 18.9% (p<0.01)<br><br>No statistically significant change (p=0.70)<br>Estimated savings of $1,056 per patient per year
Sirvent, 2016, Spain [27]	To improve the flow of critically ill patients in the intensive care unit hospital	Intensive care unit, Pre- Post, (n=1,388), 12 months	Lean	ICU boarding time (Mean)<br>Personal satisfaction (Mean)<br>Length of stay (Mean)<br>Readmissions to ICU (Percentage)<br>Emergency transfer due to lack of beds	Decreased from 360.8 to 276.7 min (p=0.036)<br>Increased from 6.6 to 7.5 (p=0.001)<br>No statistically significant change (p=0.992)<br>No statistically significant change (p=0.966)<br>Decreased from 45 to 14.3% (p=0.045)
Vose, 2014, USA [28]	To address emergency department overcrowding	Emergency department, Pre-Post, 24 months	Lean	Pull time (boarding time average)<br>Overall patient satisfaction<br>Capacity	Decreased from 58.9 to 43.6 min<br>Increased from 60-80 to 90<br>Increased 14 bed h per day
Niemeijer, 2013, Netherlands [29]	To improve efficiency of care and reducing the LOS	Department of Surgery, Pre-Post, (n= 332), 45 months	Lean Six Sigma	Length of stay (Average)<br>Duration of surgery (Average)<br>Cost saving	Decreased from 13.5 to 9.3 days (p=0.000)<br>Decreased from 154 to 98 min. (p=0.000)<br>Estimated savings of €120,000
Sorensen, 2019, USA [30]	To develop a Lean quality improvement intervention for knee and hip arthroplasty patients	Department of Surgery, Pre-Post, (n=4,253), 36 months	Lean	Length of stay (Mean)<br>30-day readmission (Percentage)<br>Discharge to home (vs rehabilitation facility or skilled nursing facility) (Percentage)<br>Patient satisfaction	Decreased from 3.2 to 2.4 (p<0.001)<br>Decreased from 3.1 to 1.1% (p=0.032)<br>Increased from 72 to 91% (p<0.001) for hip patients; Increased from 70% to 87% (p<0.001) for knee patients<br>Increased from 4.7 to 4.9 (p=0.013)

Moo-Young, 2019, USA [31]	To increase the percentage of patients discharged	Pediatric gastroenterology, Pre-Post, (n=355), 12 months	Lean Six Sigma	30-day readmission (rate)<br>Discharged before 1 pm (Percentage)<br>Length of stay (Mean)<br>Potential associated savings	No statistically significant change (p=0.54)<br>No statistically significant change<br>Decreased from 5.7 to 4.7 days (p=0.055)<br>Estimated savings of $373,000
Cerfolio, 2019, USA [32]	To improve operating room turnover time	Operating room, Pre-Post, (n=128), 6 months	Lean	Operating room turnover (Median)<br>Return of investment	Decreased from 37 to 14 min (p<0.0001)<br>Estimated return on investment of $19,500 per day
Ankrum, 2019, USA [33]	To prevent environmental transmission of pathogens	Isolation room, Pre-Post, (n=38), 2 months	Lean	Room turnover time (Median)<br>Time between room breakdown to cleaning start time (Median)<br>Room cleaning complete to UV disinfection start<br>Duration of room cleaning and curtain changing	Decreased from 130 to 65 min (p<0.0001)<br>Decreased from 10 to 3 min (p=0.004)<br>Decreased from 36 to 8 min (p<0.0001)<br>Decreased from 57 to 37 min (p<0.0001)
Peter, 2011, USA [34]	To identify possible causes for delay in performing operating procedures on time and instituting effective interventions	Operating room, Pre-Post, 24 months	Lean Six Sigma	Cases starting on time (Percentage)	Increased from 13 to 80%
Allen, 2009, USA [35]	To apply a DMAIC approach to a streamline patient discharge process at a community hospital	Hospital discharge process, Pre-Post, (n=150), 6 months	Six Sigma	Discharge time (Average)<br>Records with specific types of omissions (Percentage)	Decrease from 3.3 to 2.8 h (p=0.068)<br>Decrease of 79% in missing entries
El-Eid, 2015,	To assess the effectiveness of using Six Sigma	Emergency department, Pre-Post, (n=17,054), 10 months	Six Sigma	Discharge time (Mean)<br>Patients discharge order before noon (Percentage)	Decreased from 2.2 to 1.7 h (p<0.001)<br>No statistical change (p=0.008)

Lebanon [36]	methods to improve the patient discharge process			Patients leaving the room before noon (Percentage)<br>Hospital length of stay (Mean)<br>Length of stay of admitted ED patients (Mean)	Increased from 15.9 to 20.7% (p<0.001)<br>Decreased from 3.4 to 3.1 days (p<0.001)<br>Decreased from 6.9 to 5.9 h (p<0.001)
Vijay, 2014, India [37]	To reduce the discharge cycle time process at a tertiary care hospital	Surgical department, Pre-Post, (n=120), 3 months	Six Sigma	Cycle time of patient discharge process (Average)	Decreased from 234 to 143 min
Deldar, 2017, USA [38]	To identify etiologies of late surgery, start times, implement lean, and analyze their effects	Operating room, Pre-Post, (n=4,492), 7 months	Lean	On-time starts (Percentage)	Increased from 57 to 69% (p<0.01)
Adams, 2004, USA [39]	To decrease the mean and SD in turnaround time and to decrease the percentage of cases outside upper specification limits for General Surgery Service	Operating room, Pre-Post, (n=96), 8 months	Six Sigma	Turnaround time between cases in the operating room (Mean)	Decreased from 22.8 to 15.6 min

Note. OR indicates operating room; RIE, Rapid improvement event; ED, Emergency department; TPS, Toyota Production System; BPPI, Baystate Patient Progress Initiative; h, Hours; TS, Thoracic surgery; GYN, Gynecologic oncology surgery; Gen/CRS, General and colorectal surgery; CEGA, Cervical esophagogastric anastomotic; NSS, No statistical significance; IPOC, Interdisciplinary plan of care; CMS, Centers for Medicare and Medicaid Services; UV, Ultraviolet. Only the last name of the first author and the year of publication are shown.

Table 5. Risk of Bias.

Study	D1	D2	D3	D4	D5	D6	D7	Overall
Adams (2004)	(-)	(X)	(-)	(-)	(+)	(X)	(-)	(-)
Allen (2009)	(-)	(X)	(-)	(-)	(+)	(+)	(-)	(-)
Ankrum (2019)	(-)	(X)	(-)	(-)	(+)	(+)	(-)	(-)
Artenstein (2017)	(X)	(X)	(-)	(-)	(X)	(+)	(-)	(X)
Beck (2016)	(-)	(X)	(-)	(-)	(+)	(+)	(-)	(-)
Beck (2015)	(-)	(X)	(-)	(-)	(+)	(+)	(-)	(-)
Bender (2015)	X	(X)	(-)	(-)	(+)	(-)	(-)	(X)
Brunsman (2018)	(-)	(X)	(-)	(-)	(+)	(+)	(-)	(-)
Burkitt (2009)	(-)	(X)	(-)	(-)	(+)	(+)	(-)	(-)
Castaldi (2016)	X	(X)	(-)	(-)	(X)	(+)	(-)	(X)
Cerfolio (2019)	(-)	(X)	(-)	(-)	(+)	(+)	(-)	(-)
Cima (2011)	(-)	(X)	(-)	(-)	(+)	(+)	(-)	(-)
Collar (2012)	(-)	(X)	(-)	(-)	(+)	(+)	(-)	(-)
De la Lama (2013)	(-)	(X)	(-)	(-)	(+)	(+)	(-)	(-)
Deldar (2017)	(-)	(X)	(-)	(-)	(+)	(+)	(-)	(-)
El-Eid (2015)	(-)	(X)	(-)	(-)	(+)	(+)	(-)	(-)
Fairbanks (2007)	(-)	(X)	(-)	(-)	(X)	(-)	(-)	(X)
Gayed (2013)	(-)	(X)	(-)	(-)	(+)	(+)	(-)	(-)
Hassanain (2016)	(-)	(X)	(-)	(-)	(X)	(+)	(-)	(-)
Hseng-Long (2011)	(-)	(X)	(-)	(-)	(+)	(-)	(-)	(-)
Iannettoni (2011)	(X)	(X)	(-)	(-)	(+)	(-)	(-)	(X)
Johnson (2016)	(-)	(X)	(-)	(-)	(+)	(+)	(-)	(-)
Molla (2018)	(-)	(X)	(-)	(-)	(+)	(+)	(-)	(-)
Montella (2017)	(X)	(X)	(-)	(-)	(+)	(+)	(-)	(X)

Moo-Young (2019)	(-)	(X)	(-)	(-)	(+)	(+)	(-)	(-)
New (2016)	(-)	(X)	(-)	(-)	(+)	(+)	(-)	(-)
Niemeijer (2013)	(-)	(X)	(-)	(-)	(+)	(+)	(-)	(-)
Niemeijer (2010)	(X)	(X)	(-)	(-)	(+)	(X)	(-)	(X)
Peter (2011)	(X)	(X)	(-)	(-)	(X)	(X)	(-)	(X)
Sayeed (2018)	(-)	(X)	(-)	(-)	(+)	(+)	(-)	(-)
Singh (2014)	(X)	(X)	(-)	(-)	(+)	(+)	(-)	(X)
Sirvent (2016)	(-)	(X)	(-)	(-)	(+)	(+)	(-)	(-)
Sorensen (2019)	(X)	(X)	(-)	(-)	(+)	(+)	(-)	(X)
Tagge (2017)	(-)	(X)	(-)	(-)	(+)	(+)	(-)	(-)
Toledo (2013)	(-)	(X)	(-)	(-)	(+)	(+)	(-)	(-)
Trzeciak (2018)	(-)	(X)	(-)	(-)	(+)	(+)	(-)	(-)
Vijay (2014)	(X)	(X)	(-)	(-)	(+)	(X)	(-)	(X)
Vose (2014)	(-)	(X)	(-)	(-)	(X)	(-)	(-)	(-)
Yousri (2011)	(-)	(X)	(-)	(-)	(+)	(+)	(-)	(-)

Domains: D1: Bias due to confounding. D2: Bias due to selection of participants. D3: Bias in classification of interventions. D4: Bias due to deviations from intended intervention. D5: Bias due to missing data. D6: Bias in measurement of outcomes. D7: Bias in selection of the reported result. **Judgement:** (X) Serious. (-) Moderate. (+) Low. Only the last name of the first author and the year of publication are shown.

References

1. Iannettoni, M.D.; Lynch, W.R.; Parekh, K.R.; McLaughlin, K.A. Kaizen method for esophagectomy patients: Improved quality control, outcomes, and decreased costs. *Ann. Thorac. Surg.* **2011**, *91*, 1011–1017, doi:10.1016/j.athoracsur.2011.01.001.
2. Hseng-Long, Y.; Chin-Sen, L.; Chao-Ton, S.; Pa-Chun, W. Applying lean six sigma to improve healthcare: An empirical study. *African J. Bus. Manag.* **2011**, *5*,

12356–12370, doi:10.5897/AJBM11.1654.
3. Gayed, B.; Black, S.; Daggy, J.; Munshi, I.A. Redesigning a Joint Replacement Program using Lean Six Sigma in a Veterans Affairs Hospital. *JAMA Surg.* **2013**, *148*, 1050–1056, doi:10.1001/jamasurg.2013.3598.
4. De la Lama, J.; Fernandez, J.; Punzano, J.A.A.; Nicolas, M.; Nin, S.; Mengual, R.; Ramirez, J.A.A.; Raya, A.-L.L.; Ramos, G. Using Six Sigma tools to improve internal processes in a hospital center through three pilot projects. *Int. J. Healthc. Manag.* **2013**, *6*, 158–167, doi:10.1179/2047971913Y.0000000044.
5. Beck, M.J.; Okerblom, D.; Kumar, A.; Bandyopadhyay, S.; Scalzi, L. V. Lean intervention improves patient discharge times, improves emergency department throughput and reduces congestion. *Hosp. Pract.* **2016**, *44*, 252–259, doi:10.1080/21548331.2016.1254559.
6. Castaldi, M.; Sugano, D.; Kreps, K.; Cassidy, A.; Kaban, J. Lean philosophy and the public hospital. *Perioper. Care Oper. Room Manag.* **2016**, *3*, 25–28, doi:10.1016/j.pcorm.2016.05.006.
7. Trzeciak, S.; Mercincavage, M.; Angelini, C.; Cogliano, W.; Damuth, E.; Roberts, B.W.; Zanotti, S.; Mazzarelli, A.J. Lean Six Sigma to Reduce Intensive Care Unit Length of Stay and Costs in Prolonged Mechanical Ventilation. *J. Healthc. Qual.* **2018**, *40*, 36–43, doi:10.1097/JHQ.0000000000000075.
8. Burkitt, K.H.; Mor, M.K.; Jain, R.; Kruszewski, M.S.; McCray, E.E.; Moreland, M.E.; Muder, R.R.; Obrosky, D.S.; Sevick, M.A.; Wilson, M.A.; et al. Toyota production system quality improvement initiative improves perioperative antibiotic therapy. *Am. J. Manag. Care* **2009**, *15*, 633–642.
9. New, S.; Hadi, M.; Pickering, S.; Robertson, E.; Morgan, L.; Griffin, D.; Collins, G.; Rivero-Arias, O.; Catchpole, K.; McCulloch, P. Lean Participative Process Improvement: Outcomes and Obstacles in Trauma Orthopaedics. *PLoS One* **2016**, *11*, e0152360, doi:10.1371/journal.pone.0152360.
10. Collar, R.M.; Shuman, A.G.; Feiner, S.; McGonegal, A.K.; Heidel, N.; Duck, M.; McLean, S.A.; Billi, J.E.; Healy, D.W.; Bradford, C.R. Lean management in academic surgery. *J. Am. Coll. Surg.* **2012**, *214*, 928–936, doi:10.1016/j.jamcollsurg.2012.03.002.
11. Artenstein, A.W.; Rathlev, N.K.; Neal, D.; Townsend, V.; Vemula, M.; Goldlust, S.; Schmidt, J.; Visintainer, P.; Albert, M.; Alli, G.; et al. Decreasing Emergency Department Walkout Rate and Boarding Hours by Improving Inpatient Length of Stay. *West. J. Emerg. Med.* **2017**, *18*, 982–992, doi:10.5811/westjem.2017.7.34663.
12. Hassanain, M.; Zamakhshary, M.; Farhat, G.; Al-Badr, A. Use of Lean methodology to improve operating room efficiency in hospitals across the Kingdom of Saudi Arabia. *Int. J. Health Plann. Manage.* **2016**, *32*, 133–146, doi:10.1002/hpm.2334.
13. Yousri, T.A.; Khan, Z.; Chakrabarti, D.; Fernandes, R.; Wahab, K. Lean thinking: Can it improve the outcome of fracture neck of femur patients in a district general hospital? *Injury* **2011**, *42*, 1234–1237, doi:10.1016/j.injury.2010.11.024.
14. Montella, E.; Di Cicco, M.V.; Ferraro, A.; Centobelli, P.; Raiola, E.; Triassi, M.; Improta, G. The application of Lean Six Sigma methodology to reduce the risk of healthcare–associated infections in surgery departments. *J. Eval. Clin. Pract.* **2017**, *23*, 530–539, doi:10.1111/jep.12662.
15. Cima, R.R.; Brown, M.J.; Hebl, J.R.; Moore, R.; Rogers, J.C.; Kollengode, A.; Amstutz, G.J.; Weisbrod, C.A.; Narr, B.J.; Deschamps, C. Use of Lean and Six

Sigma Methodology to Improve Operating Room Efficiency in a High-Volume Tertiary-Care Academic Medical Center. *J. Am. Coll. Surg.* **2011**, *213*, 83–92, doi:10.1016/j.jamcollsurg.2011.02.009.

16. Singh, S.; Nair, D.; Remya, T.; Shijo, T.M.; Nair, P. Lean six sigma application in reducing nonproductive time in operation theaters. *J. Natl. Accredit. Board Hosp. Healthc. Provid.* **2014**, *1*, 1, doi:10.4103/2348-6139.132908.
17. Bender, J.S.; Nicolescu, T.O.; Hollingsworth, S.B.; Murer, K.; Wallace, K.R.; Ertl, W.J. Improving operating room efficiency via an interprofessional approach. *Am. J. Surg.* **2015**, *209*, 447–450, doi:10.1016/j.amjsurg.2014.12.007.
18. Beck, M.J.; Gosik, K. Redesigning an inpatient pediatric service using Lean to improve throughput efficiency. *J. Hosp. Med.* **2015**, *10*, 220–227, doi:10.1002/jhm.2300.
19. Tagge, E.P.; Thirumoorthi, A.S.; Lenart, J.; Garberoglio, C.; Mitchell, K.W. Improving operating room efficiency in academic children's hospital using Lean Six Sigma methodology. *J. Pediatr. Surg.* **2017**, *52*, 1040–1044, doi:10.1016/j.jpedsurg.2017.03.035.
20. Toledo, A.; Carroll, T.; Arnold, E.; Tulu, Z.; Caffey, T.; Kearns, L.; Gerber, D. Reducing liver transplant length of stay: A lean six sigma approach. *Prog. Transplant.* **2013**, *23*, 350–364, doi:10.7182/pit2013226.
21. Fairbanks, C. b. Using six sigma and lean methodologies to improve or throughput. *AORN J.* **2007**, *86*, 73–82, doi:10.1016/j.aorn.2007.06.011.
22. Molla, M.; Warren, D.S.; Stewart, S.L.; Stocking, J.; Johl, H.; Sinigayan, V. A Lean Six Sigma Quality Improvement Project Improves Timeliness of Discharge from the Hospital. *Jt. Comm. J. Qual. Patient Saf.* **2018**, *44*, 401–412, doi:10.1016/j.jcjq.2018.02.006.
23. Niemeijer, G.C.; Trip, A.; Ahaus, K.T.B.; Does, R.J.M.M.; Wendt, K.W. Quality in Trauma Care: Improving the Discharge Procedure of Patients by Means of Lean Six Sigma. *J. Trauma Inj. Infect. Crit. Care* **2010**, *69*, 614–619, doi:10.1097/TA.0b013e3181e70f90.
24. Sayeed, Z.; Anoushiravani, A.; El-Othmani, M.; Barinaga, G.; Sayeed, Y.; Cagle, P.; Saleh, K.J. Implementation of a hip fracture care pathway using lean six sigma methodology in a level I trauma center. *J. Am. Acad. Orthop. Surg.* **2018**, *26*, 881–893, doi:10.5435/JAAOS-D-16-00947.
25. Brunsman, A.C. Using lean methodology to optimize time to antibiotic administration in patients with sepsis. *Am. J. Heal. Pharm.* **2018**, *75*, S13–S23, doi:10.2146/ajhp161017.
26. Johnson, A.E.; Winner, L.; Simmons, T.; Eid, S.M.; Hody, R.; Sampedro, A.; Augustine, S.; Sylvester, C.; Parakh, K. Using Innovative Methodologies From Technology and Manufacturing Companies to Reduce Heart Failure Readmissions. *Am. J. Med. Qual.* **2016**, *31*, 272–278, doi:10.1177/1062860614562627.
27. Sirvent, J.M.; Gil, M.; Alvarez, T.; Martin, S.; Vila, N.; Colomer, M.; March, E.; Loma-Osorio, P.; Metje, T. Lean techniques to improve flow of critically ill patients in a health region with its epicenter in the intensive care unit of a reference hospital. *Med. Intensiva (English Ed.* **2016**, *40*, 266–272, doi:10.1016/j.medine.2015.08.007.
28. Vose, C.; Reichard, C.; Pool, S.; Snyder, M.; Burmeister, D. Using LEAN to improve a segment of emergency department flow. *J. Nurs. Adm.* **2014**, *44*, 558–

563, doi:10.1097/NNA.0000000000000098.
29. Niemeijer, G.C.; Flikweert, E.; Trip, A.; Does, R.J.M.M.; Ahaus, K.T.B.; Boot, A.F.; Wendt, K.W. The usefulness of lean six sigma to the development of a clinical pathway for hip fractures. *J. Eval. Clin. Pract.* **2013**, *19*, 909–14, doi:10.1111/j.1365-2753.2012.01875.x.
30. Sorensen, L.; Idemoto, L.; Streifel, J.; Williams, B.; Mecklenburg, R.; Blackmore, C. A multifaceted intervention to improve the quality of care for patients undergoing total joint arthroplasty. *BMJ Open Qual.* **2019**, *8*, e000664, doi:10.1136/bmjoq-2019-000664.
31. Moo-Young, J.A.; Sylvester, F.A.; Dancel, R.D.; Galin, S.; Troxler, H.; Bradford, K.K. Impact of a Quality Improvement Initiative to Optimize the Discharge Process of Pediatric Gastroenterology Patients at an Academic Children's Hospital. *Pediatr. Qual. Saf.* **2019**, *4*, e213, doi:10.1097/pq9.0000000000000213.
32. Cerfolio, R.J.; Ferrari-Light, D.; Ren-Fielding, C.; Fielding, G.; Perry, N.; Rabinovich, A.; Saraceni, M.; Fitzpatrick, M.; Jain, S.; Pachter, H.L. Improving Operating Room Turnover Time in a New York City Academic Hospital via Lean. *Ann. Thorac. Surg.* **2019**, *107*, 1011–1016, doi:10.1016/j.athoracsur.2018.11.071.
33. Ankrum, A.L.; Neogi, S.; Morckel, M.A.; Wilhite, A.W.; Li, Z.; Schaffzin, J.K. Reduced isolation room turnover time using Lean methodology. *Infect. Control Hosp. Epidemiol.* **2019**, *40*, 1151–1156, doi:10.1017/ice.2019.199.
34. Peter, A.; Parvathaneni, A.; Wilson, C.; Tankalavage, T.; Cheriyath, P. Wheels on Time: A Six Sigma Approach to Reduce Delay in Operating Room Starting Time. *Surg. Curr. Res.* **2011**, *01*, 1–4, doi:10.4172/2161-1076.1000102.
35. Allen, T.T.; Tseng, S.-H.H.; Swanson, K.; McClay, M.A. Improving the Hospital Discharge Process with Six Sigma Methods. *Qual. Eng.* **2009**, *22*, 13–20, doi:10.1080/08982110903344812.
36. El-Eid, G.R.; Kaddoum, R.; Tamim, H.; Hitti, E.A. Improving hospital discharge time. *Med. (United States)* **2015**, *94*, e633, doi:10.1097/MD.0000000000000633.
37. Vijay, S.A. Reducing and optimizing the cycle time of patients discharge process in a hospital using six sigma dmaic approach. *Int. J. Qual. Res.* **2014**, *8*, 169–182.
38. Deldar, R.; Soleimani, T.; Harmon, C.; Stevens, L.H.; Sood, R.; Tholpady, S.S.; Chu, M.W. Improving first case start times using Lean in an academic medical center. *Am. J. Surg.* **2017**, *213*, 991–995, doi:10.1016/j.amjsurg.2016.08.025.
39. Adams, R.; Warner, P.; Hubbard, B.; Goulding, T. Decreasing Turnaround Time between General Surgery Cases: A Six Sigma Initiative. *J. Nurs. Adm.* 2004, *34*, 140–148.
